# Molecular Regulators of Cellular Mechanoadaptation at Cell–Material Interfaces

**DOI:** 10.3389/fbioe.2020.608569

**Published:** 2020-12-08

**Authors:** Juhyeon Jo, Sama Abdi Nansa, Dong-Hwee Kim

**Affiliations:** KU-KIST Graduate School of Converging Science and Technology, Korea University, Seoul, South Korea

**Keywords:** cellular mechanobiology, mechanoadaptation, mechanotransduction, cell-materials interaction, disease associated mechanoresponses

## Abstract

Diverse essential cellular behaviors are determined by extracellular physical cues that are detected by highly orchestrated subcellular interactions with the extracellular microenvironment. To maintain the reciprocity of cellular responses and mechanical properties of the extracellular matrix, cells utilize a variety of signaling pathways that transduce biophysical stimuli to biochemical reactions. Recent advances in the micromanipulation of individual cells have shown that cellular responses to distinct physical and chemical features of the material are fundamental determinants of cellular mechanosensation and mechanotransduction. In the process of outside-in signal transduction, transmembrane protein integrins facilitate the formation of focal adhesion protein clusters that are connected to the cytoskeletal architecture and anchor the cell to the substrate. The linkers of nucleoskeleton and cytoskeleton molecular complexes, collectively termed LINC, are critical signal transducers that relay biophysical signals between the extranuclear cytoplasmic region and intranuclear nucleoplasmic region. Mechanical signals that involve cytoskeletal remodeling ultimately propagate into the nuclear envelope comprising the nuclear lamina in assistance with various nuclear membrane proteins, where nuclear mechanics play a key role in the subsequent alteration of gene expression and epigenetic modification. These intracellular mechanical signaling cues adjust cellular behaviors directly associated with mechanohomeostasis. Diverse strategies to modulate cell-material interfaces, including alteration of surface rigidity, confinement of cell adhesive region, and changes in surface topology, have been proposed to identify cellular signal transduction at the cellular and subcellular levels. In this review, we will discuss how a diversity of alterations in the physical properties of materials induce distinct cellular responses such as adhesion, migration, proliferation, differentiation, and chromosomal organization. Furthermore, the pathological relevance of misregulated cellular mechanosensation and mechanotransduction in the progression of devastating human diseases, including cardiovascular diseases, cancer, and aging, will be extensively reviewed. Understanding cellular responses to various extracellular forces is expected to provide new insights into how cellular mechanoadaptation is modulated by manipulating the mechanics of extracellular matrix and the application of these materials in clinical aspects.

## Introduction

Cells are surrounded by a complex microenvironment, and their essential functions are controlled by multiple interactions between cell-cell and cell-extracellular matrix (ECM) (Leiphart et al., 2019). Cell-cell interaction refers to direct communication between neighboring cell surfaces that occur in three well-defined architectures, such as gap junctions, tight junctions, and desmosomes, which are involved in the mechanical coupling between cells as well as signal transmission (Maitre and Heisenberg, 2013; Dhowre et al., 2015). The ECM is a three-dimensional network surrounding cells, comprising various proteins, such as collagens, fibronectin, vitronectin, and laminin (Dhowre et al., 2015; Jansen et al., 2017). Through these proteins, cells respond to changes in the mechanical properties of the extracellular environment and consequently alter cell behaviors, including adhesion, migration, proliferation, and differentiation (Jansen et al., 2015).

Cells recognize and respond to mechanical cues, which govern the mechanical homeostasis of the cell (Humphrey et al., 2014). Sensing the mechanical cues of the environment, termed cellular mechanosensing, is a process in which mechanosensitive proteins including integrins, as transmembrane receptors, perceive the mechanical signals from the micro-environment. These signals are transmitted through multi-protein complexes termed focal adhesions, which convey signals into the cell via cytoskeletal remodeling. Biophysical signals propagating along the cytoskeletal architecture transmit into the nuclear interior through the linker of nucleoskeleton and cytoskeleton (LINC) complexes, leading to the structural modification of chromatin (Martino et al., 2018).

Mechanosensation is critical to enable cells to adapt to changes in the microenvironment (McNamara et al., 2012; Cai and Heilshorn, 2014). Recent studies have shown how extracellular mechanical cues directly alter the physical properties of the nucleus, how the nucleus protects genetic material, and what happens if this system malfunctions (Leiphart et al., 2019). These studies recapitulate the critical role of the nucleus and its ability to adapt to mechanical force that ultimately regulates cellular mechano-regulation (Leiphart et al., 2019). The hallmark of transition between biophysical stimuli and biochemical signaling cascades is generally termed cellular mechanotransduction; it comprises signaling pathways, such as the Rho/Rho-associated protein kinase (Rho/ROCK) pathway, that result in cell contractility, cytoskeletal and ECM remodeling, or activation of yes-associated protein and transcriptional coactivator with the PDZ-binding motif (YAP/TAZ) that triggers the initiation of a cascade of transcription factors (Dupont et al., 2011; Mo et al., 2012; Martino et al., 2018).

Advances in microfabrication technology have enabled the microscopic manipulation of individual cells by directly controlling their micro-environments, such as matrix rigidity and topography, and direct confinement of cells (Thery, 2010; Mendes, 2013). These external mechanical conditions induce the reorganization of cellular architecture that is closely related to gene expression via the modification of the chromatin state (Miroshnikova et al., 2017). Chromatin remodeling, determined by the extent to which the DNA is packaged by the histone, switches between heterochromatin and euchromatin, is largely involved in the epigenetic regulation of cell fate (Klemm et al., 2019). Cell-material interactions facilitate the critical role of the nucleus in recognizing extracellular microenvironments and transmitting physical stress into biochemical signaling that ultimately regulates cellular mechano-regulation (Leiphart et al., 2019).

Accumulating evidences suggest that an adequate cellular response system to extracellular mechanical signals is essential to maintain normal physiology of organisms. In this review, we focus on how cell organelles sense and process extracellular mechanical cues when cells are subjected to various physical alterations in their microenvironment. Furthermore, we recapitulated the pathological relevance of various cell-material interfaces to discuss how material engineering provides therapeutic insights into human diseases.

## Cell-Extracellular Matrix Interactions

### Integrin Mediated Signal Transduction

Cells form integrin-based focal adhesions to establish structural and functional interactions with the extracellular matrix (ECM). Diverse fibrous proteins comprising the ECM interact with cells via cell surface integrin family receptors, which mediate bi-directional signal transduction between cells and their extracellular microenvironment (Sun et al., 2016b). In addition to chemical stimuli, various mechanical properties of the extracellular matrix (ECM) are involved in changes in cell behavior, including cell adhesion, migration, and differentiation through epigenetic modification (Jansen et al., 2015). Recent studies have shown that the ability of cells to interpret mechanical cues plays an important role in the modification of cellular behaviors. In particular, molecular interaction between cells and their microenvironment is critical for regulating development, tissue homeostasis, and disease progression (Changede et al., 2019).

The ECM is a three-dimensional network comprising two types of macromolecules: proteoglycans and fibrous proteins (Dhowre et al., 2015; Humphries et al., 2019), and cell-ECM adhesion and signal transmission are mainly facilitated by heterodimeric transmembrane receptor integrins (Jansen et al., 2017). Integrins linking the ECM to the intracellular cytoskeletal organizations comprise 18 types of α-subunits and 8 types of β-subunits as type I transmembrane proteins in mammals. They interact with the ECM through 24 distinct types of non-covalently linked heterodimeric combinations, which enables α and β subunits of the integrin to bind to an arginine-glycine-aspartate (RGD) motif in diverse ECM proteins such as collagen, fibronectin, and vitronectin (Kechagia et al., 2019). Integrin α- and β-subunits include an ectodomain, a transmembrane domain, and a short cytoplasmic tail. Binding of the extracellular ligands to the ectodomain or binding of the N-terminal FERM (4.1 protein, ezrin, radixin, moesin) domain of talin (talin-FERM) to motifs of β-integrin cytoplasmic tail can convert folded inactive integrins into their active form with a high affinity to bind to extracellular ligands (Bauer et al., 2019), which is consistent with the conversion of the compact bent-over conformation of integrins into an extended conformation upon talin head binding (Ye et al., 2010). Moreover, the co-activator kindlin also binds to the cytoplasmic tail of β-integrin, which results in the activation of integrins (Ma et al., 2008). In essence, talin and kindlin do not directly interact with one another, but the binding of kindlin to integrin enhances talin-mediated integrin activation (Ye et al., 2010; Calderwood et al., 2013).

Moreover, integrin activation is involved in the assembly of fibrous protein components such as fibronectin and collagen. For instance, the integrin adapter protein tensin 1, which promotes the synthesis of fibronectin fibrils by translocating the fibronectin receptor α5β1 integrin (Pankov et al., 2000), is targeted by intracellular metabolic pathways through the major metabolic sensor 5' AMP-activated protein kinase (AMPK) that negatively regulates the tensin 1 expression level. Therefore, loss of AMPK promotes tensin-mediated activation of integrins after initial activation by talin and leads to fibronectin remodeling by enhancing fibrillar adhesion formation. In addition, Kank2 protein decreases mechanical signaling through integrin to actin filaments by modulating talin-actin binding by reducing the talin rod domain affinity for actin filaments (Bachmann et al., 2019).

Integrins are directly coupled with motifs of the ECM, engaging in mechanosensation in which mechanical forces are perceived by cells. While integrins bind to ECM ligands on adjacent cell surfaces in the extracellular side, integrins coupled to a ligand, such as cyclic RGDfK peptide, cause conformational changes in the cytoplasmic side, resulting in an intracellular signaling cascade termed outside-in signaling. As such, integrins are bi-directional signaling receptors that communicate with both intracellular organelles and the microenvironment (Arnold et al., 2004). Hence, integrins as physical links between cells and the ECM mediate cell adhesion. Mechanosensing through integrin would be the first step in sensing extracellular mechanical cues and transmitting these signals into the cell by the formation of molecular clusters such as focal adhesions.

### Focal Adhesion-Dependent Cell Adhesion

Focal adhesions are fundamental force sensors of the cell and transmit mechanical signals from the extracellular matrix into the cell to regulate cell-matrix adhesion and cell migration that are directly in contact with ECM components. Thus, changes in the chemical or mechanical properties of the ECM molecules can alter cellular behaviors by remodeling the molecular composition of the focal adhesions (Martino et al., 2018).

Focal adhesions, which form adhesive contact between cells and ECM via molecular clustering of intracellular cytoskeletal protein assemblies, transmembrane protein integrins, and ligands in ECM molecules, comprise a variety of proteins including talin, vinculin, paxillin, zyxin, Ena/VASP, p130Cas, and actinins (Martino et al., 2018). The recruitment of talin to the cell membrane is critical for integrin activation, and cells that lack lipid-binding residues of talin exhibit reduced activation of integrins and disassembled focal adhesions (Chinthalapudi et al., 2018), indicating the role of talin binding to the membrane-proximal sites of integrin in regulating the activation of integrins and focal adhesions (Chinthalapudi et al., 2018).

Vinculin plays an important role in sensing extracellular mechanical stimuli. For instance, conducting an equibiaxial stretching on epithelial mammary cells enhanced molecular tension across vinculin to maintain assembled focal adhesions, which revealed that vinculin could transduce external stimuli that trigger molecular reorganization in the focal adhesions (Sigaut et al., 2018). Furthermore, actin stress fibers are subjected to a spontaneous and random cycle of thinning and recovery, where the recruitment of paxillin and zyxin, subfamilies of LIM (Lin11, Isl-1, and Mec-3) domain proteins to stress fibers assists the recovery and stabilization of actin stress fibers at the strain sites (Smith et al., 2013). These studies demonstrate that focal adhesion proteins act as molecular sensors of physical forces as well as signal transducers between the extracellular matrix and cells.

Focal adhesions recruit focal adhesion kinase (FAK) in response to mechanical stimuli (Parsons, 2003). FAK is a protein tyrosine kinase with multiple binding domains, including a C-terminal focal adhesion binding FAT domain and an N-terminal FERM homology domain flanking its tyrosine kinase domain (Zhou et al., 2015). Tensional forces developed between the FAT domain that binds to paxillin and talin and the FERM domain of FAK that binds to the cell membrane via phosphatidylinositol 4, 5-bisphosphate (PIP2), lead to phosphorylation of Tyr397, which activates FAK (Zhou et al., 2015; Bauer et al., 2019). Upon FAK activation, these molecular tensional signals are transmitted to the actin cytoskeleton, which demonstrates that FAK is a key molecular player in outside-in physical signal transduction.

Formation of focal adhesion is essential for cell adhesion and migration, which is regulated by physical factors such as the level of traction force ruled by modulating actomyosin contractility. For instance, treatment with either Rho kinase inhibitor Y-27632 or myosin II inhibitor blebbistatin in the presence of FAK, reduces phosphorylation of myosin light chain (MLC), which results in inhibited Rho-induced assembly of focal adhesion. Moreover, the inhibition of Rho kinase suppresses the recruitment of vinculin to focal adhesions and weakens cell adhesion (Dumbauld et al., 2010), indicating the role of FAK in the regulation of contractility-mediated cell adhesion.

Focal adhesions have the ability to adopt different extracellular cues by regulating their compositional changes that largely depend on actomyosin contractility, where inhibition of Rho-associated protein kinase ROCK alters the molecular combination of focal adhesion proteins including zyxin, vinculin, paxillin, and FAK (Malik-Sheriff et al., 2018). Among the focal adhesion molecular components, zyxin was most responsive to perturbation of actomyosin contractility, whereas vinculin, paxillin, and FAK remained less affected. These results indicate that individual molecular components of focal adhesion differentially respond to varying levels of mechanical tension (Malik-Sheriff et al., 2018). Hence, these studies further demonstrate that each component of focal adhesions is regulated independently to modulate their composition and assembly to perform adequately in response to different mechanical signals from the microenvironment.

### Mechanotransduction Through Cytoskeletal Remodeling

Mechanical stimuli-induced cytoskeletal remodeling such as conformational changes and/or alterations in the molecular assembly of cytoskeletons is critical to facilitate cellular mechanotransduction that regulates mechanical homeostasis of the cell. The cytoskeleton is a complex network of three main interconnecting filamentous proteins: actin filaments (F-actin), microtubules (MTs), and intermediate filaments (IFs) (Huber et al., 2015). Physical interactions are mediated within and between these three cytoskeletal networks, such as IFs and MTs in the cell interior, MTs and F-actin in the cell periphery, and IFs and F-actin mainly at the periphery of the IF network (Huber et al., 2015).

Cytoskeletal interactions are necessary for structural integrity in the process of cellular mechanoresponses mainly in sensing of substrate stiffness (Mendez et al., 2014) and transmitting applied mechanical stimuli (Pritchard et al., 2014). In particular, F-actin and IFs are generally considered the determinants of cell stiffness sensing and mechanical forces that alter their relative contributions to cytoskeletal networks. F-actin, a polymeric filament, is composed of linear polymers of G-actin monomers, which are organized into various structures, including stress fibers, cortical actin networks, surface protrusions, and the contractile ring formed during cell division (Pollard et al., 2000). In addition, MTs are polymers comprising tubulin subunits, which are heterodimers formed from two closely related globular proteins called α-tubulin and β-tubulin, tightly linked together by non-covalent bonds. F-actin and MTs are both composed of globular protein chains, and IFs are composed of strong bindings between α-helical coiled coils, which elongate long fibrous subunits. In contrast to single IFs that tend to be softer than MTs and F-actin at low strain, they can endure much larger deformations in the networked organization (Bertaud et al., 2010), which demonstrate that distinct physical properties are necessary to determine cellular dynamic features such as cell polarization and migration.

Force generation in the cell mainly depends on actin cytoskeletons that enable cell-matrix adhesion and migration. Cytoskeletal tension is transmitted to the adhesion sites by actin polymerization in assistance with motor proteins, where diverse molecular mechanisms are involved. For instance, actin filaments under tension have higher affinity for actin-based motors, e.g., myosins, further increasing the probability of binding between actin and myosin II. Cytoskeletal tension in the actin network can be induced either by active actin polymerization on the membrane via the Arp2/3 complex or by myosin II filament pulling activity (Yang et al., 2012).

To identify the potential role of mechanical forces as regulators of actin filament organization, it is essential to consider the actin filament organizations sensing the mechanical loads. A variety of F-actin-based cytoskeletal architectures are required to control cell functions. For instance, in migrating cells, lamellipodia, filopodia, and lamella are formed in the protrusion of the leading edge of the cell to generate adhesive forces at the adhesion site (Ponti et al., 2004). The lamellipodia at the leading edge of migrating cells are protrusive actin structures that are nucleated in proximity to the plasma membrane by the Arp2/3 complex (Wu et al., 2012). Filopodia contain bundles of parallel-aligned actin filaments that are nucleated by various formins, such as Daam1, and cross-linked by the actin-binding protein fascin (Aramaki et al., 2016). Lamella comprise a network of contractile transverse arcs, i.e., curved actin filament bundles, which convey contractile force to the surrounding environment through their connections with dorsal stress fibers (Hotulainen and Lappalainen, 2006). Depending on the distinct organization of F-actin based architecture, mechanical forces are transferred inside the cell, where these three key pathways for F-actin dependent mechanotransduction have been reported.

In particular, F-actin and actin-binding proteins (ABPs) lead to conformational changes under mechanical forces because the change in polymerization kinetics of ABPs can be determined by these force (Harris et al., 2018). ABPs play an important role in regulating the dynamics of the actin cytoskeleton as well as the assembly of actin filaments. For instance, they redistribute actin filaments via changes in the conformational state under mechanical load (Prochniewicz et al., 2005). They also expose the binding sites for other accessory proteins in response to mechanical load or regulate mechanical load-dependent polymerization kinetics, which ultimately alters F-actin network density and growth rates (Mueller et al., 2017).

In addition to the plus-end-tracking proteins (+TIPs) of microtubules and the minus-end-tracking proteins (–TIPs), microtubules display distinct structural and functional characteristics. +TIPs including end-binding protein 1 (EB1), kinesins, and dynein promote microtubule polymerization, and these +TIPs networks are responsible for various cellular functions such as microtubule guidance along other cytoskeletal elements, microtubule arrangement, and signal transmission. In contrast, –TIPs, including calmodulin-regulated-spectrin-associated proteins (CAMSAPs), bind to microtubules via the carboxy-terminal CKK (CAMSAP, KIAA1078, and KIAA1543) domain to control microtubule network architecture by stabilizing non-centrosomal microtubules. Therefore, CAMSAPs can be described as –TIPs that regulate the stability of microtubule minus ends in a manner dependent on minus-end polymerization (Akhmanova and Steinmetz, 2015).

The binding of MTs to actin filaments via the growing end guides the polymerization and remodeling of microtubule network; therefore, post-translational modifications of the microtubule polymerization subunit, α-tubulin, can stiffen the cytoskeletal organization that regulates cellular mechanotransduction, which ultimately alters the physical properties of the tissues in response to mechanical cues (Lyons et al., 2017). Thus highly dynamic MT networks whose density and stability are regulated by post-translational modifications (PTMs) of MTs (e.g., detyrosination, acetylation, and phosphorylation) and microtubule associated proteins (MAPs) can determine cellular mechanotransduction by controlling dynamic equilibrium in MT filament growth, assembly, and association with other cytoskeletal filaments (Song and Brady, 2015). For example, the detyrosinated α-tubulin subunit of MTs defines the mechanosensitivity of substrate compliance by stiffening the cytoskeleton. Moreover, microtubule remodeling is accelerated by dynamic stretching of the adherent cells, similar to the F-actins (Walker et al., 2020), which further demonstrates that structural interactions between F-actin and microtubules are critical in cellular mechano-regulation. Taken together, remodeling of the cytoskeleton in response to mechanical cues through diverse conformational changes is essential for mechanical homeostasis in tissues.

### Intranuclear Signal Transduction via Nucleus-Cytoskeletal Connection

The nuclear envelope (NE) separates the intranuclear organelles from the cytoplasmic environment and conveys biophysical signals between these two distinct intracellular microenvironments. The nuclear envelope comprises two lipid bilayers, the internal nuclear membrane (INM) and outer nuclear membrane (ONM), which are separated by the perinuclear space (PNS) and maintain structural or functional integrity. The molecular components of NE are physically connected to the nuclear lamina and cytoskeletons (Hieda, 2019), where LINC (Linker of Nucleoskeleton and Cytoskeleton)-mediated nucleus and cytoskeletal connection is critical for mechanotransduction, which alters mechanical force into biochemical factors to regulate outside-in signaling (Schwartz et al., 2017).

Nuclear pore complexes (NPCs) embedded in the nuclear membrane are one of the most complex protein structures that mediate bidirectional nuclear-cytoplasmic transport. NPCs comprises ~30 different types of nucleoporins (Nups), among which phenylalanine–glycine repeats containing nucleoporins (FG-Nups) contribute to overall NPC architecture (Frey and Gorlich, 2007). Nuclear mechanotransduction via NPCs is regulated by transcription factors such as yes-associated protein (YAP), which is a major downstream effector of the Hippo pathway. YAP phosphorylation is regulated by the Hippo signaling pathway with key signaling proteins, which comprise the protein kinase Hippo (Hpo) and a highly conserved group of serine/threonine kinases (Rausch and Hansen, 2020). Hippo-mediated YAP/TAZ signaling regulates various central cellular processes in tissue homeostasis, including cell proliferation, apoptosis, and stress responses (Boopathy and Hong, 2019).

Contractile actomyosin complexes comprising actin filaments and myosin motors act as central mediators between mechanical cues and Hippo-YAP signaling in various mechanotransduction environments. For instance, treatment of NIH3T3 cells placed on micropatterned substrates that control cell adhesive area, with cytochalasin D or latrunculin B, which inhibits actin polymerization, or blebbistatin and ML-7, which inhibit myosin II ATPase and myosin light-chain kinase, indicated that the nucleo-cytoplasmic localization of YAP/TAZ was regulated by the adhesive area (Wada et al., 2011). Accordingly, cells with larger spreading area exhibited enhanced formation of actin stress fibers and in turn inhibits Hippo pathway, resulting in YAP nuclear sequestration, which inhibiting either the actin polymerization or actomyosin contractility reduced the nuclear localization of YAP through disrupting the formation of actin stress fibers (Wada et al., 2011). Therefore, mechanical stimuli-induced nuclear translocation of YAP is determined by actomyosin-dependent cytoskeletal interaction (Meng et al., 2016).

Megakaryoblastic leukemia 1 (MKL1) directly binding to the serum response factor (SRF) is another member of MRTF-A family of transcription factors that regulates NPC-mediated nuclear mechanotransduction, where physical stress-induced gene expression is mediated by SRF-MKL1 co-activator complexes and regulated by inner nuclear membrane protein emerin that modulates actin dynamics (Willer and Carroll, 2017).

Nuclear transport of mechanosensitive transcription factors via NPCs largely depends on a LINC-mediated manner as observed in cells grown on a rigid matrix that requires intact LINC complexes to translocate YAP into the nucleus (Elosegui-Artola et al., 2017). The LINC complex is the molecular bridge of nuclear-cytoplasmic structural connection and comprises SUN (Sad1, UNC-84) proteins in the INM and KASH (Klarsicht, Syne-1 homology, and ANC-1) domain containing nesprins, which are spectrin-repeat proteins that localize to the ONM (Wilhelmsen et al., 2006). Molecular anchorage of the KASH domain to the intranuclear lamin proteins enables direct transfer of mechanical forces from the extracellular matrix to the nucleus via LINC complexes that are conserved nuclear envelope-spanning molecular bridges (Hodzic et al., 2004; Crisp et al., 2006).

Mammals encode five SUN proteins i.e., SUN1-5, and six KASH proteins i.e., nesprins-1-4, KASH5, and lymphocyte-restricted membrane protein (Crisp et al., 2006). SUN proteins interact with the C-terminal KASH domain of nesprins, while the N-terminal SUN domain interacts with NPCs as well as nuclear lamina (Janin et al., 2017). A meshwork of intermediate filaments (IFs) consisting of A-type and B-type lamins have been implicated in diverse cellular responses to mechanical stresses, including alteration of nuclear and cellular stiffness and morphology as well as chromatin reorganization and gene expression (Dechat et al., 2010). Therefore, silencing the expression of lamin A/C abolishes the signal pathways of mechanotransduction (Poh et al., 2012). In addition, lamins interact with inner nuclear membrane proteins such as lamin B receptor (LBR), lamina-associated polypeptides (LAPs), and emerin (Schirmer and Foisner, 2007). Emerin has signaling functions by interacting with β-catenin, which is a dual function protein involved in the regulation of cell-cell adhesion and gene transcription (Markiewicz et al., 2006). Nesprins are characterized by various N-terminal regions involved in interaction with cytoskeleton components, including actin filaments, microtubules, and intermediate filaments (Haque et al., 2006). For instance, nesprin-1/2 interacts with INM protein emerin and lamin A through a C-terminal spectrin repeats (Mellad et al., 2011), whereas nesprin-3 interacts with cytoplasmic intermediated filaments through a plectin-binding motif and nesprin-4 interacts indirectly with microtubules (Ketema et al., 2007; Roux et al., 2009). Through signaling processes within these multifunctional molecular connectors, external forces transmitted to the cytoskeleton are transferred into the nucleus to adjust cellular responses.

### Mechanoadaptation Through Chromosomal Reorganization

External physical signals via cytoskeletal remodeling propagate to intranuclear chromosomal organization, a fundamental determinant of gene expression. Therefore, extracellular cues can alter the genomic content of the cell via lamina-associated chromatin domains (LADs) in the nuclear lamina (Briand and Collas, 2020). LADs are detected at different densities on all chromosomes, enriched in the vicinity of the repressive histones H3K9me2 and H3K9me3 (Pascual-Reguant et al., 2018). In addition, the nuclear envelope and chromatin are connected at the nuclear periphery, generally considered as a repressive region with limited gene activity while NPCs are in contact with euchromatin (Briand and Collas, 2020), where NPC-associated protein Tpr regulates the establishment of heterochromatin exclusion zones (HEZs) of NPCs (Krull et al., 2010).

NPCs are involved in gene activation and repression and nucleoporins as building blocks of NPCs regulate intranuclear gene expression and post-transcriptional gene regulation (Buchwalter et al., 2019). In mammals, NPCs bind to nucleoporin-associated super-enhancers at the nuclear periphery, which results in gene transcription that ultimately determines cell functions (Ibarra et al., 2016). Nuclear basket proteins featuring stretchable gateway structures control the protein transport flux to the extent to which they open (Donnaloja et al., 2019). Accordingly, pore opening followed by association with active genes can regulate gene transcription. Moreover, chromatin compaction is altered by NUP-153 stretching and its binding to lamina through SUN1 elevates the transcription level (Donnaloja et al., 2019), which demonstrates that nucleoporin-mediated chromosomal organization is involved in the regulation of gene expression.

Nuclear lamina-associated proteins are important in connecting the nuclear lamina and chromatin by acting as chromatin readers. For example, lamin B1 receptor (LBR) binds not only to lamin B1 but also to the chromatin, which is modified by H3K9me2 and H3K9me3 via heterochromatin protein 1 (HP1) (Buchwalter et al., 2019). Force transmission through the cytoskeleton to chromatin translocates the heterochromatin from the nuclear periphery to the intranuclear spaces; therefore, changing the heterochromatin state to euchromatin is a key process in regulating gene transcription (Haque et al., 2006), highlighting the importance of cytoskeletal organization in gene expression.

Chromatin condensation is also determined by nuclear morphology and cytoskeletal organization (Keeling et al., 2017; Uhler and Shivashankar, 2018). Intracellular tension determines the shape of the nucleus and cell spreading area by modulating cytoskeletal proteins, where actin and vimentin regulate cell expansion and nuclear volume. In addition, polymerization of cytoskeletal proteins such as actin, myosin, tubulin, and vimentin is directly correlated with the size of the cell spreading area. Cell spreading enhances the assembly of actin and vimentin filaments, and the higher level of actin and vimentin assemblies reversely feature higher and lower condensation levels of chromatin, respectively (Keeling et al., 2017). In essence, actin depolymerization-induced reduction of actomyosin contractility translocates histone deacetylase 3 (HDAC3) and NF-κB transcription factor p65 into the nucleus, leading to chromatin condensation (Uhler and Shivashankar, 2017). Consistently, HDAC inhibitors have been reported to increase the expression of vimentin which inhibits the nuclear translocation of HDAC and results in chromatin decondensation (Wang et al., 2018). Furthermore, less condensed chromatin has been typically observed in cells displaying enlarged nuclei (Keeling et al., 2017).

**Figure 1:**
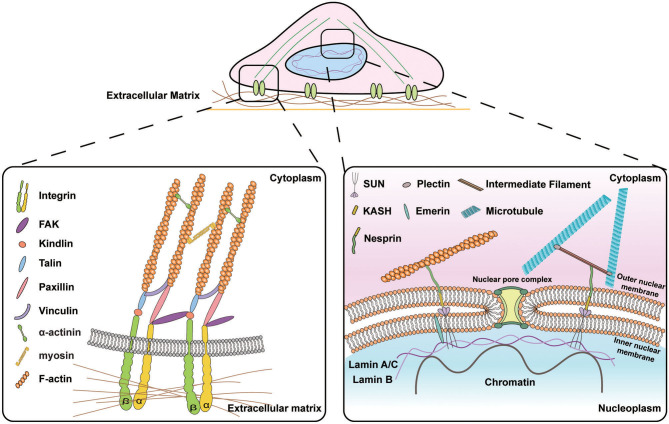
Schematic illustration of the signal transduction from the extracellular matrix (ECM) to the nucleus. (Left) Subcellular molecular machinery at the cell-materials interface. Extracellular forces applied to the cell are perceived by transmembrane protein integrins that activate the assembly of focal adhesion molecules e.g., vinculin, paxillin, and talin and recruit focal adhesion kinase (FAK) to transmit the biophysical signals into the cytoskeletons. (Right) Molecular connection between extranuclear cytoskeletons and intranuclear chromosomal organization. Transmission of biophysical signals between nucleus and cytoskeletons is mediated by linkers of nucleoskeleton and cytoskeletons (LINC) molecular complexes that are composed of outer nuclear membrane associated KASH domain proteins, e.g., nesprin isoforms that are connected to cytoskeletons in the cytoplasmic space and inner nuclear membrane associated SUN proteins that bind to lamins and chromatin in the nucleoplasmic space.

Recent studies have shown that serine/threonine-protein kinase ATR, known as ataxia telangiectasia and Rad3-related protein, mediates chromatin dynamics in response to mechanical forces (Kumar et al., 2014). The simultaneous exertion of stretching-induced mechanical stimuli on the plasma membrane or a compressive-load application on HeLa cells regulates the activation and recovery of ATR and chromatin compaction (Kumar et al., 2014). Quantitative analysis of nuclear deformations and chromatin dynamics resulting from cytoskeletal forces has further indicated that actomyosin contractility is involved in the mechanoadaption of chromatin epigenetic state in response to changes in ECM properties to maintain telomere positioning and dynamics (Makhija et al., 2016). Therefore, reduced cell-ECM physical contact partially disconnects chromatin from the nuclear membrane, resulting in elevated heterochromatin mobility (Makhija et al., 2016). Taken together, external mechanical stimuli modulate nuclear dynamics and the cytoskeleton organization, which in turn, regulate chromosomal organization by transducing the external mechanical signals through subcellular mechanoresponsive components (Figure 1), leading to alterations in gene expression by regulating the translocation of transcription factors and post-translational modifications.

## Alteration of Cell Behaviors in Response to Variation of Cell-Material Interfaces

### Principle of Cellular Functional Change: Gene Expression

Mechanical signals transduced to the nuclear membrane in assistance with actomyosin contractility induce the activation of transcription factors that promote the expression of specific genes. For instance, in response to actin polymerization as a consequence of enhanced cell polarization, myocardin-related transcription factor MRTF translocates into the nucleus in a crosstalk manner with NF-κB and binds to serum response factor (SRF) (Uhler and Shivashankar, 2017), indicating that gene expression is altered by the mechanical load. In addition, physical stimuli transmitted to chromatin via nuclear deformation determine epigenetic states (Gupta et al., 2012), which in turn results in alteration of the mechanical properties of the nucleus as well as cellular functions. In particular, higher condensation levels of chromatin decrease nuclear deformability (Kirby and Lammerding, 2018); therefore, mechanical control of chromatin condensation can be a determinant of nuclear mechanics.

Cells cultured on surfaces with different topography, geometry, and rigidity differ in cell migration, proliferation, and differentiation (Engler et al., 2006; Kilian et al., 2010) as a consequence of altered gene expression. For example, the analysis of gene expression via DNA microarrays of mesenchymal stem cells (MSCs) on diverse shapes has shown a distinct elevation of transcripts, i.e., cells on shapes with concave edges resembling star shapes displayed higher osteogenic transcripts, while adipogenic transcripts were dominant in cells on shapes without sharp edges and corners (Kilian et al., 2010). In essence, confinement of cells on star shapes increased cytoskeletal contractility (James et al., 2008); therefore, these cells exhibited elevated gene expression specifically involved in the regulation of cell tensional state, including p38, extracellular related kinase ERK1/2, c-Jun N-terminal kinase JNK, canonical, and non-canonical Wnt transcripts, and Wnt downstream regulator Rho/ROCK, which promote osteogenesis in cells. Compared to the osteogenesis of cells cultured in higher contractility-promoting shapes, cells cultured in contractility reducing shapes or unpatterned surfaces were triggered to adipogenesis (Arnsdorf et al., 2009; Kilian et al., 2010), which together indicates how geometric cues of the substrate direct cellular differentiation via altering gene expression.

Matrix stiffness-dependent phenotypic change is also determined by chromatin accessibility of transcription factors. Breast epithelial cell line, MCF10A, encapsulated in interpenetrating networks (IPNs) comprising reconstituted basement membrane and alginate, mimicking *in vivo* 3D tissue conditions, altered chromatin accessibility throughout the genome, and promoted tumorigenic phenotype (Stowers et al., 2019). It has also been reported that emerin regulates heterochromatin compaction in an HDAC3-mediated manner, by binding to HDAC3 in response to mechanical strain (Le et al., 2016; Qi et al., 2016; Stowers et al., 2019). Accordingly, the rigid matrix enhances accessible sites in Sp1-binding motifs containing chromatin through the Sp1–HDAC3/8 pathway and elevates the activity of the Sp1 transcription factor, which results in the upregulation of tumorigenic genes associated with the breast malignant neoplasm in rigid tissues (Stowers et al., 2019). In addition, mechanoresponsive gene expression can further determine cell proliferation; therefore, cyclic stretch of vascular smooth muscle cells could enhance proliferation accompanied by reduced expression of emerin and lamin A/C, which binds to DNA segments involved in the regulation of cell proliferation (Qi et al., 2016). These results suggest that matrix rigidity mechanically alters the expression of nuclear proteins including emerin and lamin A/C, which in turn regulates tumorigenesis through epigenetic modification.

Topographic characteristics of the substrate are another physical setting that regulate gene expression. Compared to unpatterned mesenchymal stem cells, cells placed on parallel grooves suppress the activity of histone deacetylase (HDAC) due to the alteration of nucleocytoplasmic transfer of HDAC as a consequence of nuclear elongation (Li et al., 2011). Microgrooved surfaces have also been utilized for cell reprogramming because they can increase di- and tri-methylation of histone H3 at lysine 4, i.e., H3K4me2 and H3K4me3 in non-transduced mouse fibroblasts and fibroblasts infected with OSKM, co-expressed transient transcription factors comprising Oct4, Sox2, Klf4, and c-Myc, which are involved in cell reprogramming (Downing et al., 2013). These results demonstrate that topographical alteration activates genetic reprogramming genes through histone modification. Treatment with blebbistatin, a non-muscle myosin-II inhibitor, disrupts actin-myosin contractility that diminishes surface topography dependence; therefore, it is highly suggested that the mechanical modulation of histone modifications is tightly regulated by cytoskeletal tension (Downing et al., 2013).

Hence, chromatin structure undergoes an epigenetic modification in response to the extracellular signals in the microenvironment to enable cells to exhibit an optimal cellular function in response to signals. These signals include matrix rigidity, topographical alteration, and extracellular forces such as strain and shear stress, which direct epigenetic responses of the cell.

### Environmental Sensing and Cell Adhesion

To interact with extracellular microenvironments, focal adhesions (FAs) are in contact with extracellular matrices through the transmembrane protein integrins, which enables the remodeling of the cytoskeleton and the transmission of various signals in response to varying features of extracellular settings. As the biochemical signaling hub of the cell-ECM interactions, the molecular composition of focal adhesions responds to the properties of the ECM recognized by integrin coupling (Starr and Fridolfsson, 2010). Mechanosensing of the extracellular microenvironment enables cells to respond to variations of cell-material interfaces such as rigidity and microstructures that alter intracellular signaling pathways through cell adhesion receptors and cytoskeletal reorganization. Hence, the physical features that the cells can sense modify biological processes such as cell migration and proliferation via cell-material interaction.

Substrate stiffness is an important mediator of cell functions such as migration, proliferation, and differentiation. Alteration of matrix stiffness has an impact on the activation of integrins and focal adhesion assembly as mechanosensors and mechanotransducers. The formation of integrin-mediated cell-matrix adhesions leads to the assembly of integrin adhesome at the adhesion site (Kanchanawong et al., 2010). The adhesion force-bearing proteins such as talin are stretched by cellular adhesion to a rigid substrate, which directly activates its binding protein, vinculin (Del Rio et al., 2009; Stutchbury et al., 2017). For instance, stretch-induced unfolding of talin can activate vinculin recruitment on stiff matrices (Margadant et al., 2011). In contrast, soft matrix can reduce the expression of β1 integrin and Caveolin-1 (Cav1), integral membrane proteins involved in integrin-dependent signaling through FAK and Src, which deregulates matrix stiffness-dependent integrin activation (Goetz et al., 2008; Yeh et al., 2017).

Geometrical and mechanical constraints on the extracellular microenvironment also alter cellular mechanosensing signaling pathways. ECM-micropatterned surfaces that primarily restrict cell adhesion and spreading have been utilized to alter cellular mechanosensation in *in vitro* conditions (Thery, 2010). To adapt to the geometry of their micropatterned environment, cells alter their attachment to confined space by readjusting cytoskeletal tension and focal adhesion organization that further changes cell adhesion and spreading (Ermis et al., 2018). For example, adhesion area-controlled human mesenchymal stem cells were differently differentiated by controlling focal adhesion assembly, where cells micropatterned on large fibronectin islands treated with transforming growth factor-beta (TGF-β) induced expression of SMC contractile markers such as alpha-smooth muscle actin (α-SMA), whereas cells on small fibronectin islands induced chondrogenesis (Wang et al., 2016).

Myosin motor protein interacts with F-actin to determine cell contractility. Cells with large adhesion areas have filament-like myosin structures, while those placed on varying micropatterns with small adhesion area show punctate myosin formation in cells due to the lack of myosin binding to F-actin, which regulates cell contractility (Albert and Schwarz, 2014). To identify cell mechanical state that primarily relies on traction force that myosin can generate along with actin filaments, atomic force microscopy (AFM) was applied to measure cytoskeletal tension (Rape et al., 2011). The MSCs adhered to the micro-patterned fibronectin-coated tissue culture polystyrene (TCPS) showed filament-like myosin aggregation in association with the ventral stress fibers and transverse arcs in cells with large adhesion area, where myosin movement along the F-actin could generate traction force (Wang et al., 2016).

By confirming the increased Young's modulus of MSCs according to the increased adhesion area, the generation of traction force based on myosin bound to F-actin can determine mechanoresponsive cellular behaviors such as cell migration and division (Vicente-Manzanares et al., 2009; Wang et al., 2016). The formation of focal adhesion clusters depends on the molecular phosphorylation, which largely relies on the adhesion size. For instance, differentiated rat embryonic fibroblasts cultured on fabricated islets by printing poly(L-lysine), grafted with poly(ethylene glycol) (PLL-g-PEG) to confine the organization of focal adhesions in a limited size has shown reduced phosphorylation of paxillin and FAK (Goffin et al., 2006). Moreover, in myofibroblasts cultured on 10- and 20-μm-long islets, β1 integrin, tensin and paxillin in supermature focal adhesions were localized at the cell periphery (Goffin et al., 2006). In contrast, in cells cultured on 2–6-μm-long islets, redistribution of the tensin and β1 integrin from the cell periphery to the cell center is induced, which also occurs when substrate stiffness is decreased (Goffin et al., 2006), where the classical focal adhesion markers β3 integrin and vinculin remained specifically in the cell periphery and paxillin was partly redistributed to the cell center, and also remained in peripheral focal adhesions. Similarly, this redistribution of matrix adhesion components occurred after switching to a culture substrate with a lower rigidity. In addition, the level of protein tyrosine phosphorylation in super mature focal adhesion was significantly higher than that in classical focal adhesion islets, and the phosphorylation of paxillin and FAK decreased significantly from the total extracts of myofibroblasts cultured on small islets (Goffin et al., 2006). These results demonstrate that adhesion size controls the phosphorylation and molecular composition of focal adhesions in a molecular tension-dependent manner.

Taken together, the molecular basis of focal adhesion-mediated mechanosensing is involved in recognizing and transferring mechanical signals generated in the extracellular environment. Clustering of integrin receptors with other related proteins leads to the formation of focal adhesions, which can perceive extracellular stimuli through molecular cascades involving the cytoskeletal reorganization that triggers outside-in signaling. Therefore, the manipulation of substrate physical conditions has fundamental roles in regulating cell behaviors by controlling these mechanoresponsive molecules and cytoskeletal remodeling, which ultimately alters cellular adhesion.

### Modulation of Cell Migration in Response to Mechanical Forces

Cell migration is critical to a variety of biological processes such as development (Fujimori et al., 2019), immune response (Luster et al., 2005), wound healing (Leoni et al., 2015), tissue regeneration (Qu et al., 2019), and cancer metastasis (Yamada and Sixt, 2019). Cell migration consists of continuous mechanosensation of extracellular microenvironments; therefore, altering the physical properties of the substrate, for example, rigidity, ligand distribution, topology, and geometry, directly regulates cell migration, which is a critical process in mechanoadaptation (Charras and Sahai, 2014; van Helvert et al., 2018).

Among the diverse *in vitro* culture methods that have been developed to control cell migration, in a fracture model that mimics fracture healing, osteoblast-like cells MG-63 and human mesenchymal stem cells (hMSCs) were also cultured on microgrooved polycaprolactone substrates to investigate the effect of surface topography on migration capacity via a wound healing assay. This study depicted the effect of microgrooves on wound gap healing, where parallel grooves with varied depth on the substrate promoted the migration of hMSCs and collective migration of MG-63 cells, and cell migration was accelerated in shallow grooves compared to deep grooves. These results reveal that controlling the physical setting of the cells could promote adequate wound healing process (Zhang et al., 2015).

The recognition of substrate geometry is particularly important to determine cancer cell malignancy; therefore, an *in vitro* model of microgrooved polydimethylsiloxane has been developed (Kushiro et al., 2017). In this model, by comparing cell motility between cancerous and non-cancerous epithelial cells originating from diverse tissues, it has been shown that cancerous cells are more motile but less sensitive to topographic variations due to insufficient formation of stable and oriented actin fibers on the grooved surface. Consequently, attenuated recognition of surface topology enhanced the metastatic potential of cancer cells, which further demonstrates the ability of cancer cells to migrate less affected by geometric hindrance in their microenvironment (Kushiro et al., 2017). These results further suggest that different sensitivity of cancer cells to such mechanical properties of the surface and exhibition of distinct cell motility must be taken into account to develop cancer therapy.

In a combined approach to modulate the stiffness of the substrate through topographical changes, a set of micropillars with oval cross-sections was designed to obtain an anisotropic stiffness that guided epithelial cells to translocate toward rigid micropillars, which was a consequence of the alignment of focal adhesions and F-actins in the same direction (Saez et al., 2007). Considering these data, molecular regulators of cellular mechanoadaptation including focal adhesions along with cytoskeletal components modulate cell motility in response to microenvironmental changes.

In addition to the topography of the substrate, ligand distribution on various surfaces is critical to regulate cell migration. For instance, mimicking the distribution of ligands via a stretchable hydrogel substrate, patterned by nanoarrays in a quasi-hexagonally arrangement, exhibited increased cell migration when the ligand spacing was enlarged and inhibited the formation of focal adhesions (Deng et al., 2017). Consistent with these results, substrate ligand presentation of ECM proteins including fibronectin, laminin, and collagen as adhesive ligands in combination with stiffness of the substrate, so called stiffness-by-ligand regulation, induced different cellular responses to stiffness such as differential focal adhesion assembly and cytoskeletal remodeling to substrate stiffness, which are dependent on the ECM ligands (Sazonova et al., 2015). To further investigate the influence of stiffness and ECM composition on cell migration, a 2D *in vitro* model was applied to mimic the blood vessel wall by culturing vascular smooth muscle cells on an elastic polyacrylamide hydrogel with tunable stiffness coated with fibronectin and collagen I (Rickel et al., 2020). Measuring the mean squared displacement of these cells exhibited increased migration distance and speed as a consequence of decreased alignment of cortical stress fibers and actin cytoskeleton disorganization in stiffer fibronectin-coated substrates in contrast to cells on collagen I-coated substrates with diminished migration distance in response to stiffness (Rickel et al., 2020). In addition, adult neural stem cells were cultured on an adhesive poly D-lysine-coated surface with laminin, a major ECM proteins in a microenvironment for neural stem cells, stripe-printed on this surface to investigate the role of ECM protein micropatterning on the migration of these cells (Joo et al., 2015). This study indicated that despite the random distribution of cells on the substrate, cells exhibited a higher affinity to migrate on poly D-lysine surfaces to move toward laminin-patterned lines (Joo et al., 2015). Hence, ECM composition in combination with physical properties of the substrate governs cell migration speed and direction by switching between distinct pathways and molecular regulators.

In addition, directed cell migration requires asymmetric protrusions enriched with RNA, where localized RNAs within the protrusions are controlled by the adenomatous polyposis coli (APC) protein that is involved in cell adhesion and migration through the regulation of β-catenin and its interaction with E-cadherin (Preitner et al., 2014). Moreover, increased migration was a result of enhanced localization of these RNAs at protrusive regions that are modulated by tuning the stiffness of the ECM as a result of increased contractility of actomyosin and Rho GTPase signaling on stiff substrates, leading to the formation of a network of microtubules that are post-translationally detyrosinated (Wang et al., 2017).

Together, these studies propose mechanical force-mediated approaches to regulate cell migration, which is a hallmark of a variety of cellular functions by modulating the physical properties of the substrate. Cell migration is an essential step in a wide range of diseases, particularly in cancer, where the regulation of cell motility by engineering the surface materials would be a promising therapeutic approach to treat these diseases.

### Mechanical Control of Cell Proliferation

The extracellular microenvironment guides cellular behaviors by a combination of signaling pathways. Changes in geometric and mechanical constraints have been shown to interfere with not only the cell structure, but also the fundamental cell fates, such as cell differentiation and proliferation. Recently, diverse approaches to manipulate cell fate have been developed by modulating the mechanical properties of the extracellular environment.

For example, the spreading area of the epidermal cells was controlled by varying the substrate stiffness, where the cells grown on soft silicone substrate displayed reduced spreading area-dependent growth i.e., growth arrest, compared to cells placed on rigid substrates (Wang et al., 2012), and various cellular features, such as focal adhesion assembly and phosphorylation of myosin light chain, were also diminished on soft substrates (Aratyn-Schaus and Gardel, 2010). These cells also showed a reduction in proliferation and migratory potential, as well as the formation of focal adhesions; therefore, these observations recapitulate the effect of reduced actomyosin contractility on the suppression of cell proliferation in the compliant microenvironment (Mih et al., 2012). Furthermore, analysis of the proliferative potency assessed by the fractional change in the synthesis phase of a cell cycle, indicated that cell proliferation increased along with the increase in substrate stiffness only in some specific cancer cell lines (Tilghman et al., 2010). For instance, among these rigidity-dependent cell lines, lung carcinoma cell line A549 cultured on a soft collagen-coated polyacrylamide hydrogel showed increased E-cadherin expression level and defect in the G1/S checkpoint of cell cycle as a consequence of lack of adhesion signaling. This accumulation of cells in G1 phase on soft gels could be a consequence of induced apoptosis that inhibits cell growth (Tilghman et al., 2010). In addition, A549 cell line showed significant increase in cell spreading on rigid substrate, indicating that the extent of cell proliferation correlates with ability of cells to spread on substrates with different rigidity. Together, on rigid substrates, increased FAK activation controls adhesion signaling and increases spreading of these cells which in turn regulates proliferation rate. Interestingly, A549 cells seeded into the lungs of nude mice did not form detectable micro colonies, which is consistent with the results of the lower growth rate of these cells on soft gels that represented the rigidity level similar to lung tissue (Tilghman et al., 2010). Hence, these results recapitulate the correlation of cell's ability to grow on these rigidity-tunable substrates to the *in vivo* physical properties of tissues.

In addition, as fibroblasts cultured on flat surface prefer rigid matrix, cyclic stretching of soft substrates filled with PDMS nanopillars could mimic stiff substrates and thus induced elevated cell proliferation and spreading compared to unstretched condition, where nuclear translocation and accumulation of MRTF-A and YAP were detected. Accordingly, depletion of MRTF-A on soft nanopillar substrates suppressed the translocation of YAP into the nucleus, and blocked stretch-dependent cell spreading and growth. Similarly, YAP-depleted cells on soft nanopillars did not exhibited nuclear translocation of MRTF-A, and failed to proliferate. These results further indicate that cyclic stretch-induced translocation of MRTF-A and YAP to the nucleus is required to stimulate the cell spreading and proliferation on compliant matrix (Cui et al., 2015).

Micropatterning techniques have also been used to confine the cell geometry that regulates cell proliferation (Thery, 2010) because engineered micropatterns could provide an effective way to investigate the sensitivity of cell responses to diverse extracellular microenvironments. Primary rat mesenchymal stem cells confined onto circular micro-islands of arginine-glycine-aspartate (RGD) on poly(ethylene glycol) (PEG) substrates (Peng et al., 2011), as the cell-confining adhesive area increased, the fraction of proliferating cells increased (Yao et al., 2019). Conversely, cells cultured on small micro-islands inhibited cell spreading and induced chromatin condensation that reduced DNA synthesis, which is attributed to restricted cell proliferation (Versaevel et al., 2012).

Furthermore, micro-grooved surfaces can be applied to control cell proliferation (Watt and Huck, 2013). MSCs cultured on micro-scaled grooved surfaces showed a higher proliferation rate with a higher percentage of cells in the S/G2-M-phase than standard culture flask-grown cells (Chaudhary and Rath, 2017). In the case of the application of fibronectin (FN)-immobilized micro-grooved titanium surface that enables the unique combination of nanoscale, sub-microscale, and microscale topography and the cell adhesive ECM molecules, proliferation of human gingival fibroblasts was enhanced, where focal adhesion proteins c-Src, FAK, integrin-linked kinase (ILK), and downstream signaling molecules such as c-Fos, c-Fyn, c-Jun, and c-Myc were significantly upregulated (Kim et al., 2018). Therefore, the alteration of substrate rigidity and topography or cell shape confinement can promote cell proliferation by modifying cell adhesion through modulating a diversity of mechanoresponsive components including focal adhesions and transmembrane protein integrins.

### Mechanoresponsive Cell Differentiation

To maintain mechanical homeostasis, stem cells adjust their lineage commitment to various external mechanical cues such as substrate physical properties or external forces including stretching, compression, and tensional forces (Steward and Kelly, 2015; He et al., 2018). A variety of studies have confirmed that mesenchymal stem cells incubated on soft (0.1–1 kPa), moderately stiff (11 kPa), and stiff (34 kPa) matrices exhibited an enhancement in the expression of neurogenic, myogenic, and osteogenic transcripts, respectively (Engler et al., 2006). Disruption of actomyosin contractility of these cells inhibited the expression of lineage markers; therefore, alternative stem cell differentiation on varying substrate rigidity depends on non-muscle myosin II (NMM II)-based cytoskeletal tension (Engler et al., 2006), which is determined by nuclear mechanosensation of substrate stiffness (Swift et al., 2013).

In agreement with these studies, a single-well platform consisting of polyacrylamide hydrogels with gradients of linear stiffness was applied to observe cellular behavior over a wide range of stiffness changes (Hadden et al., 2017). In this model, human mesenchymal stem cells expressed more MRTF-A, a transcription factor involved in myogenic differentiation, myogenic transcription factor MyoD, and osteogenic marker CBFA1 on gels with a stiffness of ~20, 12, and 36 kPa, respectively, which mimics various tissue stiffness levels including muscle and bone (Hadden et al., 2017). In addition, to investigate the effect of stiffness on the differentiation of tendon-derived stem cells, the expression of a wide range of tenogenic, adipogenic, chondrogenic, and osteogenic transcription markers was observed in gelatin hydrogels with varying matrix stiffness (Liu et al., 2018). Quantitative analysis based on real time PCR has shown that cells cultured on compliant substrates preferred tenogenesis, chondrogenesis, and osteogenesis rather than myogenesis, which is primarily regulated via the FAK-ERK1/2 pathway (Liu et al., 2018). Consistent with these results, performing qRT-PCR revealed that on stiff substrates of ~62–68 kPa, osteogenic differentiation is promoted as a consequence of upregulated integrin α5 and its downstream signaling pathways including FAK, p-ERK, and p-Akt (Hamidouche et al., 2009; Sun et al., 2018). Moreover, the expression of β-catenin and phosphorylated GSK-3β were reported to be elevated on stiff substrates, and as the FAK/Akt pathway is known to regulate the expression of GSK-3β, suggesting a mediating role of this pathway in regulating differentiation in response to substrate rigidity (Fang et al., 2000; Sun et al., 2018).

Alteration of cell morphology has been a challenge to control stem cell differentiation. Rho GTPases are known to be critical for the differential fate of cells, whose activation is mediated by insulin-like growth factor 1 (IGF-1) and Rac1 through phosphorylation and subcellular localization of Rho regulator, Rho GTPase activating protein (RhoGAP) (Taya et al., 2001; Sordella et al., 2003; Bustos et al., 2008). Human mesenchymal stem cells cultured on fibronectin-coated islands controlling the cell shape regulate differentiation via the activation of Rho/ROCK signaling (McBeath et al., 2004). In addition, cell shape modulates the spreading area and in turn regulates the cytoskeleton tension; therefore, higher and lower levels of RhoA activation were reported to promote osteogenic and adipogenic differentiation, respectively (McBeath et al., 2004). This is consistent with cell shape-dependent adipo/osteogenesis via the MAPK signaling pathway (Kilian et al., 2010). The human osteosarcoma cell line MG-63 was also cultured on microgrooved polystyrene substrates with diverse width and distance between individual grooves (Sun et al., 2016a). As shown in mRNA transcript analysis, in this model, cells on substrates with narrow grooves and ridges resulting in cells with a larger spreading area expressed enhanced osteogenic markers (Sun et al., 2016a). Hence, topographical changes in the cell-contacting materials modulate cell differentiation by altering molecular pathways such as Rho/ROCK and MAPK, resulting in distinct expression of differentiation markers.

It has also been reported that the activity of YAP/TAZ transcription factor regulates the shifting between the differentiation of mammary epithelial multipotent progenitors to either myoepithelial cells (MEP) or luminal epithelial cells (LEP) (Pelissier et al., 2014). In addition, to observe the role of substrate stiffness and geometry on the activity and localization of transcription factor YAP/TAZ, mammary epithelial cells and MSCs were cultured on fibronectin-coated polyacrylamide hydrogels with diverse rigidity and micropatterned islands of varying size, where soft substrates and small islands promoted cytoplasmic localization of YAP/TAZ, whereas its nuclear localization was dominant on stiff substrates and enlarged islands due to elevated stress fiber formation and Rho activation because cytoskeletal tension is known to regulate YAP/TAZ in parallel to the Hippo pathway (Dupont et al., 2011; Dobrokhotov et al., 2018; Dasgupta and McCollum, 2019). Thus, rigid substrates promote YAP/TAZ activity and consequently promote differentiation to MEP relative to LEP (Pelissier et al., 2014), which indicates that the MEP/LEP specification is regulated by the mechanical properties of the substrate.

Integrin-mediated mechanotransduction is also modulated by magnetically triggered dynamic ligand-presenting hydrogels, where bioactive ligands of ECM proteins, for example, fibronectin, were differentially presented on soft matrices (Wong et al., 2020). In this platform, hyaluronic acid hydrogel was conjugated with Arg-Gly-Asp-bearing magnetic nanoparticle (RGD-MNP), and the concealing or promotion of the RGD-MNP presentation was controlled by the directional magnetic attraction, which could inhibit or induce differentiation potential of hMSCs (Wong et al., 2020). For instance, exposing the RGD ligands through upward magnetic attraction promotes the adhesion of cells to the surface via increasing focal adhesion formation, and in turn, regulating the differentiation by increasing the mechanical load sensed by cells (Wong et al., 2020).

These models suggest a new approach to *in vivo* tissue regeneration by the application of stem cells through modulating the interaction of cells with ECM to define cell differentiation. These approaches mainly rely on alteration of the actomyosin contractility and molecular regulators of cellular mechanoadaptation, which in turn, regulating mechanoresponsive pathways such as FAK-ERK1/2, Rho/ROCK, and YAP/TAZ.

**Figure 2:**
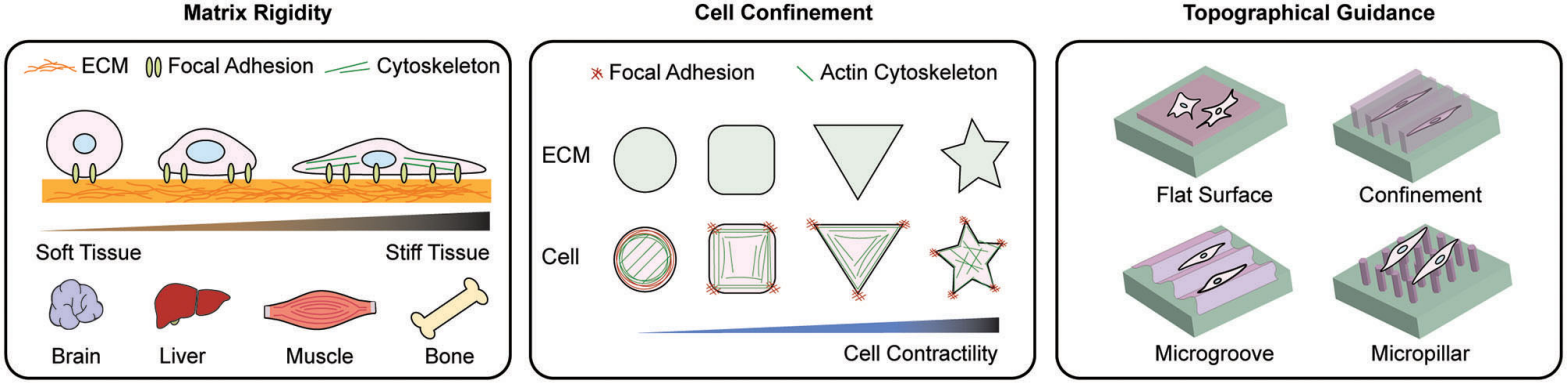
Scheme of representative materials engineering to modulate mechanoadaptation. Manipulation of matrix rigidity, morphological confinement, and topographical guidance have been applied to regulate mechanoresponse at cell-material interface. To study the effect of rigidity on cellular behaviors, various ECM stiffness levels relevant to *in vivo* tissue stiffness are applied. By controlling the cell morphology cell spreading dynamics and the reorganization of intracellular organelles are explored. Topographical manipulation of the cell-materials interface guides cell alignment, adhesion, and migration. These approaches induce distinct cellular behaviors including cell adhesion, migration, proliferation and differentiation.

**Figure 3:**
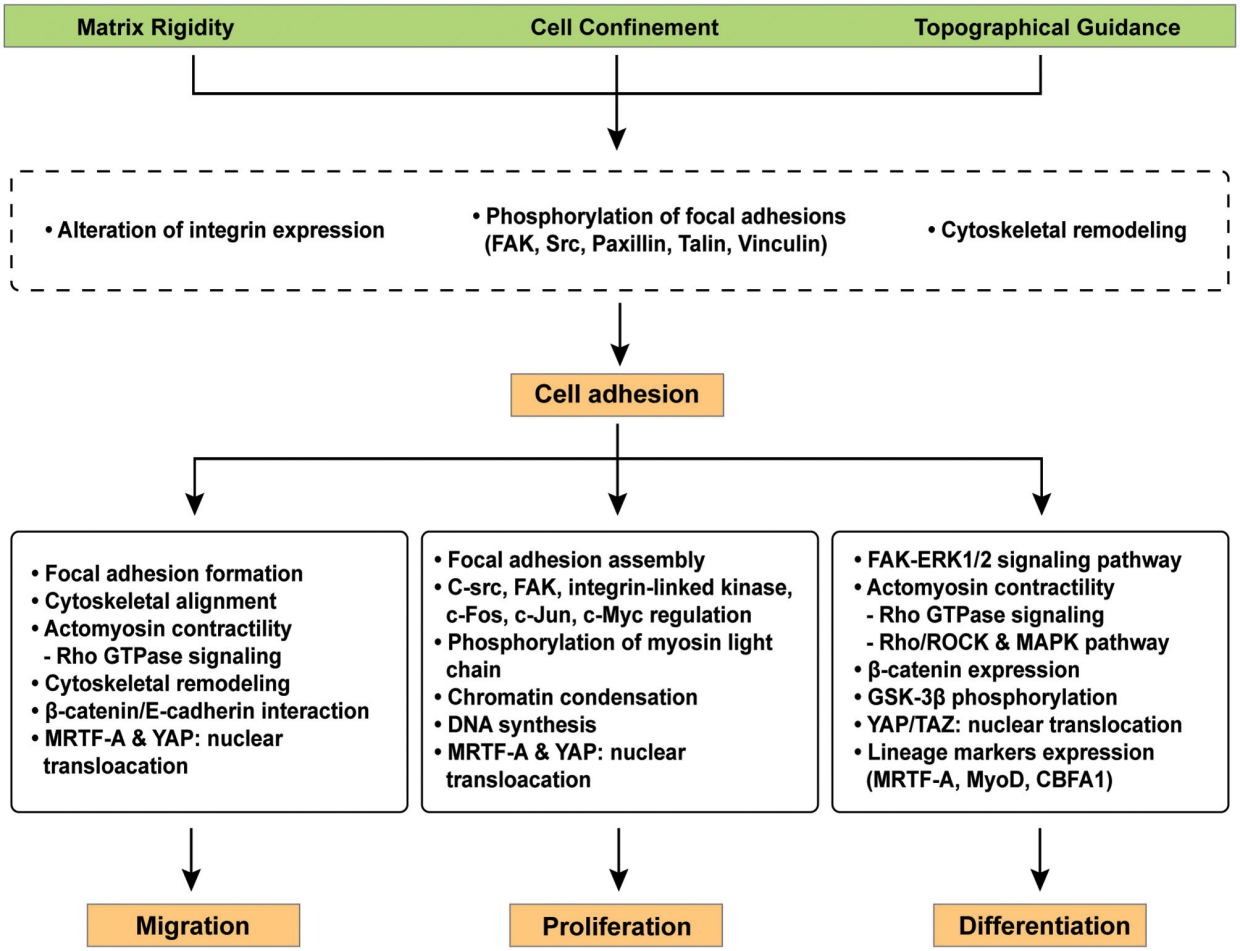
Representative signaling pathways involved in cellular mechano-regulation at the cell-material interface. Various surface modifications adjust critical cell behaviors e.g., migration, proliferation, and differentiation in the field of tissue engineering because cellular interaction with extracellular microenvironment at the cell-materials interface facilitates complex signaling cascades. Mechano-regulated signaling pathways facilitates the reorganization of focal adhesions, cytoskeletal remodeling, and/or cell contractility via the expression of distinct genes.

Since cell differentiation is largely altered by cell adhesion and spreading, therefore, recent advances in manipulation of cell-material interfaces (Figure 2) recapitulate the underlying mechanism of cellular mechanoadaptation to the extracellular microenvironment (Figure 3).

## Disease Relevance of Cellular Mechanoresponse at Cell-Material Interfaces

### Cardiovascular Disease

**Figure 4:**
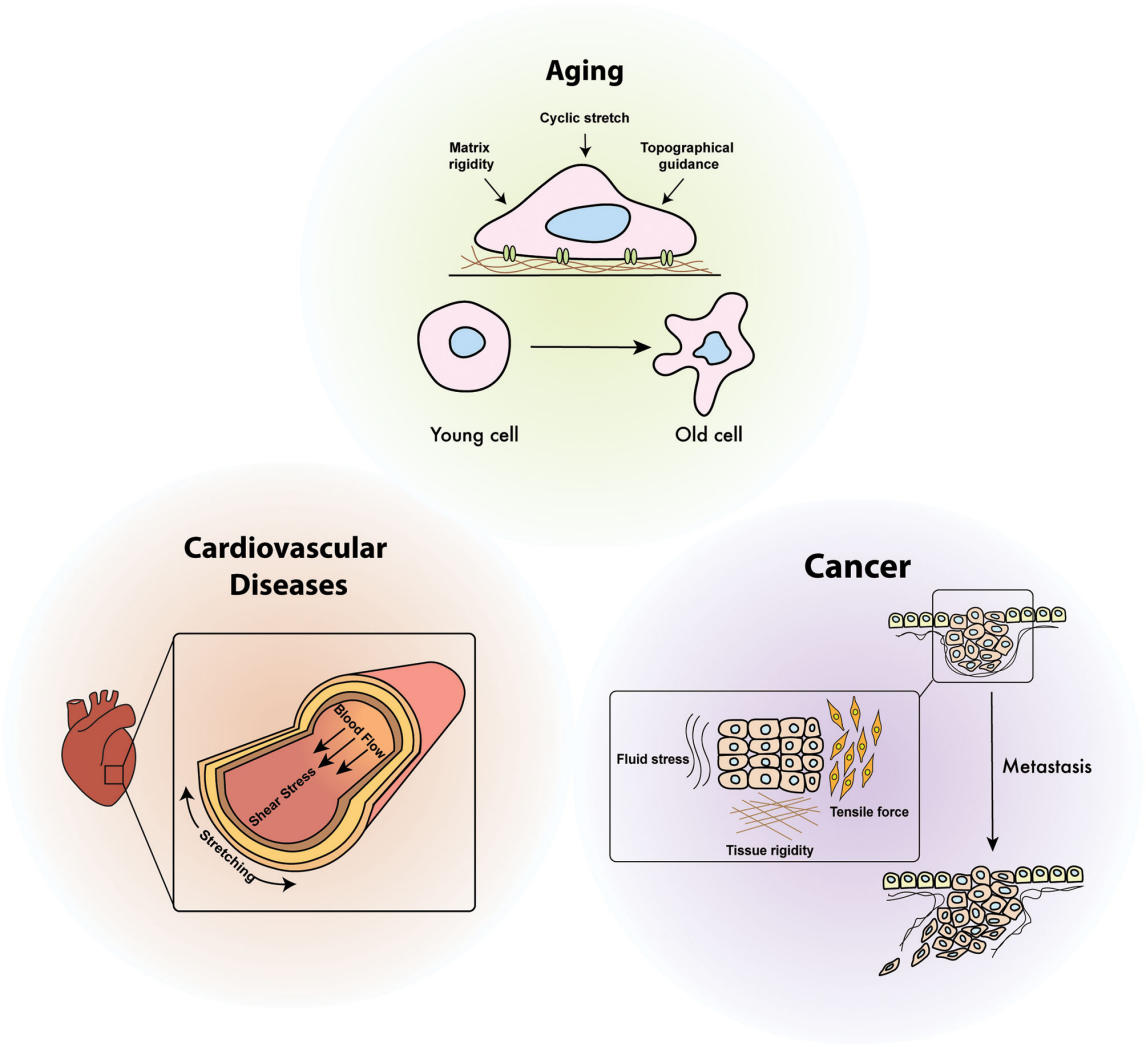
Diseases relevance of cellular mechanoresponses. Cellular mechano-regulation is essential to maintain mechanical homeostasis in response to various mechanical stimuli including shear stress, cyclic stretch, environmental stiffening, and tensile force. Defects in mechanical signaling pathways due to continuously generated physical stimuli inside the organ are attributed to the onset of diverse human diseases such as aging, cancer, and cardiovascular diseases.

Since mechanical stimuli sensed by cells are transmitted through intracellular signaling transduction pathways, defect in these signaling pathways result in altered physiological responses or pathological progression (Figure 1). Cardiovascular cells are typically exposed to a variety of mechanical stimuli, including blood pressure and wall shear stress, and abnormal responses to these stimuli cause lethal heart diseases such as hypertension, aortic reflux, and myocardial infarction (Dostal et al., 2014) (Figure 4). While the role of mechanical stimuli has been recognized for the developmental biological processes and morphogenesis of the cardiovascular system, the cellular response to mechanical cues is now emerging as a major pathophysiological determinant in the diagnosis of heart failure (Garoffolo and Pesce, 2019).

The mechanical interaction between ECM and cells play a critical role in cardiac differentiation and maturation during heart morphogenesis (Garoffolo and Pesce, 2019). For example, an increase in matrix rigidity of the heart primordium is associated with the initial beating of embryonic cardiomyocytes because matrix stiffness regulates the coordinated opening of the mechanosensitive Ca^2+^ channels before electromechanical coupling begins (Chiou et al., 2016). Cardiomyocytes that acts as a contractile unit of the heart muscle tissue generate contractile wave through their contractions by mechanical strain, where high strains propagate the Ca^2+^ wave, leading to cardiomyocyte contraction. This indicates that the first signaling pathways for the induction of coordinated myocyte beating in the early embryonic heart tube is based on mechanical stimuli and these mechanical forces are associated with the strengthening of the heart matrix (Chiou et al., 2016). Furthermore, differentiation of cardiomyocytes followed by adaptation to extracellular environment involves considerable rearrangement of contractile structures (Bildyug, 2019). The stiffness of ECM can determine the rearrangement of the contractile apparatus of cardiomyocytes because the cells placed on the rigid polyacrylamide hydrogels coated with the type I collagen formed unaligned sarcomeres and stress fiber-like structures in myofibril organization, in contrast to cardiomyocytes cultured on a compliant matrix similar to the stiffness of the myocardium (Bildyug, 2019). In addition, cells cultured on soft substrates exhibit reduced contraction force, while cells cease to contract on stiff substrates (Bhana et al., 2010). Therefore, the reduction in tissue stiffness by pharmacological inhibitors of ECM remodeling (e.g., β-aminopropionitrile, BAPN) or increase with exogenous crosslinkers (e.g., ribose) can stimulate heart regeneration and cardiomyocyte proliferation (Wang et al., 2020). The BAPN, an ECM remodeling inhibitor, can reduce tissue stiffness by irreversible inhibition of lysyl oxidase, an enzyme that combines collagen and elastin with the ECM. Ribose has also been used to enhance the stiffness of collagen hydrogel *in vitro* as a crosslinker through glycan (Levental et al., 2009). Based on these results, reverse-differentiated mammalian cardiomyocytes can reactivate cell cycles by reducing matrix rigidity, and cardiac regeneration accompanied by temporary softening of the extracellular matrix has been achieved (Yahalom-Ronen et al., 2015; Yu et al., 2018).

Cardiovascular cells are accompanied by subtle changes in pro-inflammatory/pro-fibrotic phenotypes depending on the biophysical characteristics of the surrounding matrix(Garoffolo and Pesce, 2019). Pro-fibrotic activity on cardiac fibroblasts in the failing heart, for instance, is highly associated with metabolic insults, inflammatory stimuli, and epigenetic changes, which has been reported by previous studies showing that mesenchymal stem cells have “mechanical memory” effects(Yang et al., 2014). In particular, mesenchymal stem cells cultured on soft poly (ethylene glycol) (PEG) hydrogels showed deactivation of YAP/TAZ as well as the expression of pre-osteogenic transcription factor RUNX2, which was determined by pre-incubation time on a rigid polystyrene culture plate. Furthermore, mesenchymal stem cells cultured on TCPS (mechanical dosing) for a short period of time led to reversible YAP activation (Yang et al., 2014). However, constitutive activation of YAP occurs through the threshold dose even after the removal of the mechanical dose, which is termed as the mechanical memory effect (Yang et al., 2014). In addition, without mechanical dosing, mesenchymal stem cells are differentiated toward adipogenic and osteogenic lineages but mechanical dose prior to culturing on soft hydrogel induces differentiation toward osteogenesis with increased RUNX2 expression. Thus, similar to epigenetic memory associated with the exposure of cells to changed metabolic conditions, tissue mechanics can also alter the expression of pathology-associated genes even after returning matrix mechanics to normal conditions (Vinci et al., 2013).

The importance of mechanical stimuli such as shear stress and cyclic strain has also been recapitulated by *in vitro* cardiac differentiation. Mouse embryonic stem cells cultured on collagen type IV-coated glass slides with steady laminar shear stress applied by a fluid flow bioreactor promoted differentiation toward ectodermal (< 0.5 N/m^2^) or mesodermal (0.5 or 1.5 N/m^2^) lineage depending on the magnitude and duration of shear stress (Wolfe et al., 2012). Moreover, embryonic stem cell-derived cardiomyocytes cultured on elastic poly (lactide-co-caprolactone) (PLCL) scaffolds subjected to cyclic strain sowed elevated cardiac-specific gene expression compared to non-stretched cells (Gwak et al., 2008). Therefore, application of shear stress and cyclic stretch during cardiac differentiation can be a complementary approach for tissue engineering to combat cardiovascular cell-associated dysfunctions.

In summary, cardiovascular cells are typically exposed to a variety of mechanical stimuli ranging from shear stress to compression. Furthermore, these cells recognize the mechanical properties of the surrounding matrix including the stiffness, shear stress, and cyclic strain to modify intracellular signaling, which results in the pathogenesis of the cardiovascular system. Accordingly, mechanical regulation of cell responses is essential during cardiac development, and an understanding of these mechano-regulation mechanisms has the potential to reverse the developmental deficiency. Ultimately, these mechanisms will establish a new route to treat cardiovascular disorders by controlling cell mechanics-dependent intracellular signal pathways.

### Cancer

As shown in a variety of biological processes and disease progression, mechanical signals transmitted from the microenvironment play a key role in tumorigenesis and cancer metastasis (Figure 4). Expansion and growth of the tumor mass, enhanced interstitial pressure, and altered intracellular contractility are attributed to intracellular forces in cancer; therefore, the manipulation of extracellular mechanical stimuli can be a promising therapeutic approach to inhibit cancer progression (Yu et al., 2011).

Tumor stiffness depends on the intratumoral amount of ECM proteins such as hyaluronan and collagen (Gkretsi and Stylianopoulos, 2018). Mathematical modeling revealed that the displacement and growth of solid tumors such as breast cancer and colon adenocarcinoma tumors from their host tissues require higher stiffness levels than their surrounding tissues (Voutouri et al., 2014), indicating the role of tumor stiffness in cancer progression. Recent studies have further shown that ECM stiffness is a critical determinant of cancer metastasis that directly promotes invasion and migration capacities by controlling the actomyosin contractility, which promotes the assembly of invadosomes and lamellae (Kai et al., 2016). In essence, ECM stiffness triggers an enhancement in integrin clustering and the formation of focal adhesions, which in turn activates Rac and Cdc42 GTPase activity required for the assembly of invadosomes and lamella via assembly of the FAK and steroid receptor coactivator (FAK-Src) complex (Kai et al., 2016).

Colorectal cancer biophysical model of CCD18 stromal cells, induced to express cancer associated fibroblast (CAF) phenotype via TGF-β treatment, have shown elevated secretion of activin A in case that they were cultured on rigid fibronectin matrix, which in turn, led to enhanced migration and epithelial to mesenchymal transition (EMT) (Staudacher et al., 2017; Bauer et al., 2020). In addition, this model proposed that stiffness levels elevated up to 40 kPa, representing colorectal cancer tumor stiffness, results in the highest secretion level of activin A (Bauer et al., 2020).

Clinically, it is known that rigid and dense breast tissues manifest an enhanced risk of metastasis and cancer progression (Yu et al., 2011). The activation of yes-associated protein (YAP) in response to enhanced substrate rigidity accelerates the motility of diverse cancer cells, where activated YAP accumulates in the nucleus and acts as a critical upstream regulatory protein of MMP-7 that induces an elevated level of integrin β1, integrin α2, and epidermal growth factor receptor (EGFR). Therefore, enhanced cell proliferation on stiff substrates can accelerate the viability of cells (Nukuda et al., 2015). In addition, the possibility of increased proliferation and migratory potential in non-transformed mammary epithelial cells in the absence of stromal cells was reported to be dependent on the activation of the MAPK signaling pathway via elevated Rho expression in response to enhanced stiffness (Provenzano et al., 2009). In addition, stiffer matrices were reported to induce migration and invasion capacity of the human salivary adenoid cystic carcinoma cell line ACC2, which was a consequence of enhanced actin filament organization paralleled with up- and down-regulation of matrix metalloproteinases (MMPs) and tissue inhibitor of matrix metalloproteinases (TIMPs) activity, respectively, in response to the increased activation of the RhoA/ROCK pathway (Zhao et al., 2018). Moreover, stiff matrices trigger the expression of Sp1 through the Sp1–HDAC3/8 pathway, which results in tumor progression (Stowers et al., 2019). In a recent study comparing a variety of cancer cell lines placed on rigid or compliant fibronectin-coated pillars, cancer cells were less contractile on the rigid matrix, indicating the disruption of rigidity-sensing cytoskeletal proteins in cancer cells (Yang et al., 2020). In addition, using a 3D matrigel platform with varying stiffness revealed that EMT was promoted on stiffer matrices as a consequence of nuclear translocation of the EMT transcription factor TWIST1, which is released from its cytoplasmic anchoring protein partner Ras GTPase-activating protein-binding protein 2 (G3BP2), which they bind via the conserved G3BP2-motif in TWIST1 proteins of vertebrates (Wei et al., 2015). These results confirm that mechanosensing of matrix compliance by cancer cells is critical for determining cancer progression.

Viscoelasticity of the extracellular microenvironment has been recently introduced as another critical determinant in tumor progression. Viscosity of tumor assessed by magnetic resonance elastography (MRE) showed that malignant tumors are more dependent on the power law than benign tumors, which indicated that malignant lesions display a viscous and fluid-like property compared to benign tumors (Sinkus et al., 2007). Viscosity and stiffness of the ECM has similar effects on alteration of signaling pathways involved in mechanoadaptation. For instance, enhanced viscosity of ECM by changing its composition triggers YAP/TAZ signaling (Bennett et al., 2018; Papalazarou et al., 2018). This behavior can be explained based on the molecular clutch model, where higher viscosity promotes higher force loading rate and thus inducing nuclear translocation of YAP that activates its downstream pathways (Bennett et al., 2018). These induce cell proliferation and results in increased tumorigenic phenotype.

EMT is a hallmark of cancer metastasis that can be modulated by not only the stiffness of the surrounding tissues but also the geometric features that cancer cells can sense. In mouse mammary epithelial cells cultured on 2D epithelial sheets of defined size and shape, including square, rectangular, and sinusoidal shapes, treatment with TGF-β exhibited that EMT was spatially expressed higher on the corners and edges of the square-shaped fibronectin-coated island regions due to nuclear localization of SRF cofactor MRTF-A (Gomez et al., 2010). In essence, the localization of MRTF-A is regulated by the dynamics of the actin cytoskeleton, which itself is modulated in a Rho-dependent manner and in response to the isometric tension within a tissue, in which higher tension enhances MRTF-A nuclear localization, interpreting the high expression of EMT at edges and corners, perceiving higher loads of mechanical stress (Nelson et al., 2005; Fan et al., 2007; Zhao et al., 2007; Gomez et al., 2010). In addition, changing the square shapes to rectangular and sinusoidal shapes to modulate the gradient of mechanical stress showed higher expression of the mesenchymal marker α-smooth muscle actin (αSMA) and reduced the expression of epithelial marker cytokeratin at the short edges and convex regions of sinusoidal monolayers, respectively (Gomez et al., 2010). This implies that substrate geometry regulates the load of mechanical forces perceived by cells and thus can modulate critical behavior of cancer cells such as EMT.

Manipulation of the materials property to simulate tumor characteristics *in vitro* provides an insight into future therapeutic approaches of cancer. For instance, biomaterials engineering to control tissue stiffness, geometry, and ligand distribution can be a novel approach to enhance anti-cancer drug delivery efficacy (Lin et al., 2019) and develop a drug screening platform for the efficient treatment of cancers (Grolman et al., 2015). Moreover, a photo-activated biomaterial-conjugated cell penetrating peptide (CPPs) was introduced to temporally and spatially control cellular attachment and cellular growth upon photo activation (Lin et al., 2019). This photo switchable cell–biomaterial interface exhibited an enhanced potential of cellular uptake of biomolecules and nanomaterials upon light activation, which can control the delivery of drugs to decrease the side effects of toxic drugs and increase the efficiency of therapies, proposing an applicable model for *in vivo* studies.

The emergence of tissue engineering in cancer therapy is to modulate the malignancy potential of tumors and/or the delivery of chemotherapeutic reagents into tumors, which can find a novel therapeutic approaches into cancers.

### Aging

Aging is a multifaceted chronological process accompanied by biochemical and biophysical modification of individual cells and their surrounding microenvironment (Phillip et al., 2015), which induces epigenetic modification and alteration of overall cell mechanics that will ultimately adjust biophysical interactions between intracellular organelles and the extracellular microenvironment (Park et al., 2020). Biological aging of an organism typically involves failure of cellular functions. In particular, cellular senescence resulting from the deterioration of cell division and uncontrolled proliferation, generally termed cancer, has been identified as a lethal consequence of the aging process (Campisi, 2013), where modified ECM composition and matrix stiffening is attributed to the loss of mechanical integrity between cells and their microenvironment (Phillip et al., 2015). ECM remodeling is an important feature in the development, morphogenesis, and tissue regeneration; therefore, cellular aging should be considered within the context of cell-ECM interaction (Figure 4).

Since changes in the mechanical properties of cells and their interaction with changing microenvironment are typical characteristics of the aging process (Starodubtseva, 2011), diverse approaches have been challenged to identify statistical relationships between biological age and cell mechanics. For instance, atomic force microscopy analysis of cytoplasmic deformability of adherent human epithelial cells and cardiomyocytes showed differential mechanosensation of substrate rigidity in response to biological aging (Berdyyeva et al., 2004; Lieber et al., 2004). Experiments conducted using a hydrogel that mimics the stiffness of myofibers have further demonstrated that the aging-induced elevated stiffness of myofibers suppressed the activation/proliferation of myogenic progenitor cells (Lacraz et al., 2015). Moreover, the regenerative capacity of oligodendrocyte progenitor cells adult CNS progenitor cells declined in an age-dependent manner (Sim et al., 2002), which was attributed to the loss of cellular differentiation resulting from microenvironmental stiffening. These cells regained their regenerative capacity by inhibiting PIEZO1, the mechanoresponsive ion channel, by placing them onto a soft environment (Segel et al., 2019), hence, biological aging can be a critical determinant of cell-matrix interaction.

To determine how aging alters cellular response to extracellular mechanical cues, mechanical stimulation was applied to differentially aged cells. In response to uniaxial cyclic strain, for instance, human foreskin fibroblasts from older donors reoriented faster than the fibroblasts from younger donors due to age-dependent changes in the actin cytoskeleton (Zahn et al., 2011). This stress-responsive cellular realignment is associated with the reorientation of the actin stress fibers that are largely aligned predominantly perpendicular to the stretching direction (Greiner et al., 2013). Based on these results, cellular aging can lead to altered cell mechanical properties through cytoskeletal remodeling, which defines fundamental cell physiology.

Recent studies have identified that aging is attributed to dysregulated immune/inflammatory reactions that determine pro-inflammatory states (Accardi and Caruso, 2018), where the endoplasmic retina (ER) stress is considered as a potential regulator of age-related inflammation (Salminen et al., 2010). Since ER is a complex membrane network that is located adjacent to the nuclear envelope and extends throughout the cytoplasm, it can detect and transmit biochemical signals from diverse subcellular compartments (Naidoo, 2009). Altered ER homeostasis, such as disruption of adequate protein folding and/or accumulation of unfolded proteins, results in apoptosis and inflammation that are implicated as hallmarks of various aging-associated pathological progressions (Naidoo, 2009). Biological aging increases ER stress that mediates pro-inflammatory responses and mechanical stimuli induce ER stress (Valentine et al., 2018; Salminen et al., 2020); therefore, age-dependency once to the mechanical stretch has been studied in the context of inflammatory responses. Specifically, alveolar type II cells obtained from young and old C57BL6/J mice were exposed to cyclic stretch to mechanically stimulated cells (Phillip et al., 2015), where ER stress-induced inflammation and monocyte recruitment were more dominant in aged cells (Valentine et al., 2018).

Material engineering approaches such as micropatterning can alter age-dependent phenotype of cells through modifying cytoskeletal organization and its linkage to the nucleus. For instance, Hutchinson-Gilford Progeria Syndrome (HGPS) results in rapid and premature aging caused by mutation in *Lmna* gene, encoding nuclear major structural proteins lamin A and C. Mutation in this gene leads to the expression of truncated pre-lamin A, referred to as progerin (Dechat et al., 2007). Smooth muscle cells derived from HGPS induced pluripotent stem cells (HGPS-iPSC-SMCs) were compared on micropatterned PDMS with grooves and ridges with flat substrates (Pitrez et al., 2018). These results exhibited an increased expression of various aging-related markers including progerin in HGPS-iPSC-SMCs on micropatterned surfaces compared to flat substrates. In addition, these cells showed higher alignment of their nuclei on micropatterned substrates along the major axis of micropatterned surfaces, leading to higher disruption of LINC complex and in turn promotes the accumulation of progerin through DNA damage. Thus, investigating the biophysical interaction in cell-engineered materials interfaces will provide a novel insight into the development of implantable devices incorporating human tissues, which are in a long-term contact with tissues to avoid accelerated cellular aging.

Altogether, age-dependent phenotypic alteration and progression of chronic diseases can be characterized by biophysical features of individual cells that vary with aging, and inversely, cellular mechanoresponses to extracellular physical conditions could help to predict the progression of aging processes. Thus, understanding the connections between these biophysical cellular mechanisms and the evolution of chronic diseases will be a breakthrough with regard to strategies aimed at rejuvenating organs subjected to aging-induced damage.

## Discussion

The mechanism by which cells recognize and transmit extracellular mechanical forces is a highly sophisticated process involving a variety of proteins in diverse subcellular compartments. Modulating tissue geometry, stiffness, and biophysical properties results in the perception of different loads of mechanical signals from the microenvironment. It is important that modulation must be based on a good understanding of different parameters in compliance with *in vivo* conditions and their effect on cellular behavior. In addition, the combined effect of multiple biophysical and biochemical stimuli must be considered to analyze the results of a specific model system. For instance, a piezoelectric fibrous scaffold, which enabled tunable topography from micropatterns to fibrous bundle structures, can be an approach to the modulation of assembly of neuron-specific cytoskeletal proteins, which in turn can regulate YAP translocation into the nucleus (Kim et al., 2020). These results can provide a new approach for neural-related tissue engineering and neural implantation by modulating the differentiation of neural cells through topographical cues and alteration of mechanoresponsive pathways. Furthermore, it has been proposed that in contrast to 2D studies of YAP translocation as one the main mechanotransducing factors in cells, in 3D models mimicking *in vivo* conditions, stiffness-mediated progression of breast cancer is not YAP-dependent (Lee et al., 2019). These results emphasize the need to utilize an appropriate model to study the mechanical properties of tissues and moreover, while the overall complexity of mechanotransduction in 3D microenvironments has been less identified, in order to facilitate our understanding of cellular behaviors in *in vivo* condition, we should not be restrained from accepting additional dimensions.

For the practical application in the field of bone tissue regeneration, physical properties of the extracellular microenvironment are crucial to design a load-bearing implant that is ideal for the regeneration of large bone defects. Recently proposed scaffold model is to mimic the *in vivo* bone ingrowth in response to mechanical loads (Entezari et al., 2020). This device was introduced as a model to support a wide range of hydrogel- and ceramic-based scaffolds with a capability of responding to various mechanical stimuli by controlling the level of mechanical strain and stress. These studies highlight the necessity of comprehensive models in the field of mechanobiology to better study the interaction of cells with their surrounding materials. Herein, we recapitulate the importance of such emerging approaches in tissue regeneration and disease therapy. Thus, establishment of a proper *in vivo* mimicking models considering diverse physical and chemical factors is critical to introduce reliable models for practically efficient clinical application. We expect our review could provide noble insights into the cellular mechanoadaptation and its relevance to human diseases.

## Author Contributions

JJ and SA co-wrote the manuscript. D-HK supervised the project and co-wrote the manuscript. All authors contributed to the article and approved the submitted version.

## Conflict of Interest

The authors declare that the research was conducted in the absence of any commercial or financial relationships that could be construed as a potential conflict of interest.
